# Toughening, Reinforcing, and Reprocessing of Epoxy Resin with Hyperbranched Polymer Containing Disulfide and Imine Dual Dynamic Covalent Bonds

**DOI:** 10.3390/polym18121418

**Published:** 2026-06-06

**Authors:** Xu Sun, Chen He, Yan Zhang

**Affiliations:** Key Laboratory of Specially Functional Polymeric Materials and Related Technology (Ministry of Education), School of Materials Science and Engineering, East China University of Science and Technology, Shanghai 200237, China; y30230778@mail.ecust.edu.cn (X.S.); y82240304@mail.ecust.edu.cn (C.H.)

**Keywords:** hyperbranched polymers, toughening, reinforcing, dual dynamic bonds, reprocessing

## Abstract

Epoxy resins are extensively utilized in various fields for their excellent comprehensive performance. However, the inherent brittleness and lack of reprocessing ability greatly limit their sustainability. In order to obtain reprocess ability in epoxy resin with superior mechanical properties and thermal stability, a curing agent (VA) and a hyperbranched epoxy toughening agent (HVT) containing disulfide and imine bonds have been synthesized from vanillin. Owing to the distinctive topological structure and abundant epoxy terminal groups of HVT, the modified epoxy resin (5HVT/E51/VA) exhibits high toughness, enhanced mechanical strength, and favorable thermal stability. When compared to the properties of the unmodified resin, the impact and flexural strength of 5HVT/E51/VA are increased by 55.32% and 71.63%, respectively. Its glass transition temperature (*T*_g_) and 5% weight loss temperature (*T*_d5%_) are also enhanced by 4.74% and 11.33%, respectively. Moreover, the resins are highly stable in most solvents, but can be completely degraded in hexylamine/2-mercaptoethanol (HAE/2-ME) solution within 2.5 h. The resin also displays notable scratch-healing capability, and the healing efficiencies reach above 85%. Even after three reprocessing cycles, their strength retention rate exceeds 80%, suggesting excellent sustainability potential. This research provides a sustainable method for preparing high-performance epoxy resins, suggesting their potential applications in self-healing and reprocessable composites.

## 1. Introduction

Epoxy resins are broadly used in the fields of transportation and aerospace due to their outstanding chemical resistance, dimensional stability, and mechanical strength [1,2]. However, the permanent cross-linked network of epoxy resin makes the reprocessing, repair, and degradation very difficult, leading to significant environmental pollution and resource waste [3,4,5]. Moreover, their inherent brittleness often causes premature failure under mechanical stress. Therefore, it is quite meaningful to develop high-performance epoxy resins with excellent reprocessing and improved toughness.

Dynamic covalent bonds, such as imine [6,7], ester [8], disulfide [9], and boronic ester [10], can undergo reversible breaking and reformation [11,12]. These bonds have been incorporated into the cross-linked network to modify the reprocessing of epoxy resins [13,14,15,16,17,18]. For example, a self-curing epoxy resin (VEP) with imine bonds was synthesized from vanillin [19]. The resulting resin could be degraded in an acidic THF/H_2_O solution at 50 °C, and reprocessed by hot pressing. However, the time for complete degradation requires two days, and a reprocessing temperature of as high as 200 °C. A tetrafunctional epoxy resin (DFBPA-EP) containing dynamic disulfide bonds was prepared from diphenolic acid and cystamine [20]. It can rapidly degrade in a dithiothreitol/dimethylformamide (DTT/DMF) solution at 80 °C within 2.5 h. However, the thermal properties of the resin are unsatisfactory, with *T*_d5%_ of only 258 °C. It is still a great challenge for epoxy resins to be quickly degraded or reprocessed under relatively mild conditions and to remain good thermal stability simultaneously.

In addition, the presence of the rigid aromatic units and the highly cross-linked three-dimensional network of epoxy resin not only endow it with high properties, but also lead to inherent brittleness [21,22,23]. Various toughening agents, such as rubber particles, thermoplastics, and nanofillers, have been used to modify epoxy resins [24,25,26,27]. For instance, a 342% enhancement in the impact strength of resin is achieved by the addition of the 5% liquid rubber (CTBN) and 15% phenolphthalein poly(aryl ether ketone) [28]. However, CTBN is usually less compatible with the resin. Recently, a hyperbranched polymer (HBP) has aroused much interest as an additive due to its unique topological structure [29,30,31]. Moreover, abundant functional groups of the HBP, such as hydroxyl, epoxy, and amino groups can participate in the curing reaction, thus modifying compatibility with the matrix [32,33,34]. For instance, the tensile strength is increased by 139.6% by the presence of 20 wt% epoxy-terminated hyperbranched polyether (EHBPE) in the epoxy blend [35]. However, the flexible aliphatic segments of the EHBPE result in a lower *T*_g_ for the blend compared to that of the unmodified resin, decreasing from 127.5 °C to 117.9 °C. By incorporating 4 wt% hyperbranched polymers (HBPDR-DPP) synthesized from resveratrol, the impact and flexural strength of the blend are increased by 83.4% and 54.6%, respectively, with respect to properties for the unmodified epoxy blend [36]. However, the cross-link density of the epoxy resin is also reduced by incorporation of HBPDR-DPP, leading to a lower *T*_g_. It is very difficult for epoxy resins to have both excellent thermal stability and toughness.

To obtain excellent reprocessing ability, mechanical strength, and thermal stability, a curing agent (VA) and a hyperbranched epoxy toughening agent (HVT) containing imine and disulfide bonds have been synthesized from vanillin. Bio-based vanillin was chosen as the raw material because its rigid aromatic ring is beneficial for enhancing the thermal stability and mechanical strength of the resin, and its aldehyde group can react with 2,2′-dithiodianiline to form imine bonds and introduce disulfide bonds. The incorporation of the highly branched structure and flexible segments of HVT greatly improves the toughness of the diglycidyl ether of bisphenol-A (E51) resin. Further, the modified resin exhibits thermal stability and mechanical strength due to the presence of aromatic units in the main chain and the abundant epoxy and hydroxyl terminal groups of HVT. Disulfide and imine bonds can be broken and reformed under various conditions. The cured 5HVT/E51/VA resin is fully degraded in hexylamine/2-mercaptoethanol (HAE/2-ME) solution just in 2.5 h. Furthermore, the resin shows remarkable scratch-healing capability, and can be reprocessed at 150 °C, with high flexural and impact strength retention rates. The resulting epoxy resin exhibits superior thermal stability, mechanical strength, and reprocessing ability, indicating its application prospects in high-performance and recyclable composites.

## 2. Materials and Methods

### 2.1. Materials

Diglycidyl ether of bisphenol-A (E51, epoxy equivalent weight of 195 g·eq^−1^) was obtained from Shanghai Aotun Chemical Technology Co., Ltd. (Shanghai, China). Vanillin (VAN, 99.0%), while 2,2′-dithiodianiline (2-AFD, 98.0%), ethyl alcohol (EtOH, 99.5%), and cystamine dihydrochloride (Cys-HCl, 99.0%) were supplied by Titan Scientific Co., Ltd. (Shanghai, China). Epichlorohydrin (ECH, 99.5%), dimethyl sulfoxide (DMSO, 99.0%), 1,1,1-tris-(hydroxymethyl)propane (TMP, 99.0%), and benzyltriethylammonium chloride (TEBAC, 99.0%) were purchased from Aladdin Co., Ltd. (Shanghai, China). All chemicals were used as received without further purification.

### 2.2. Synthesis of Vanillin-Based Curing Agent (VA)

**Figure 1:**
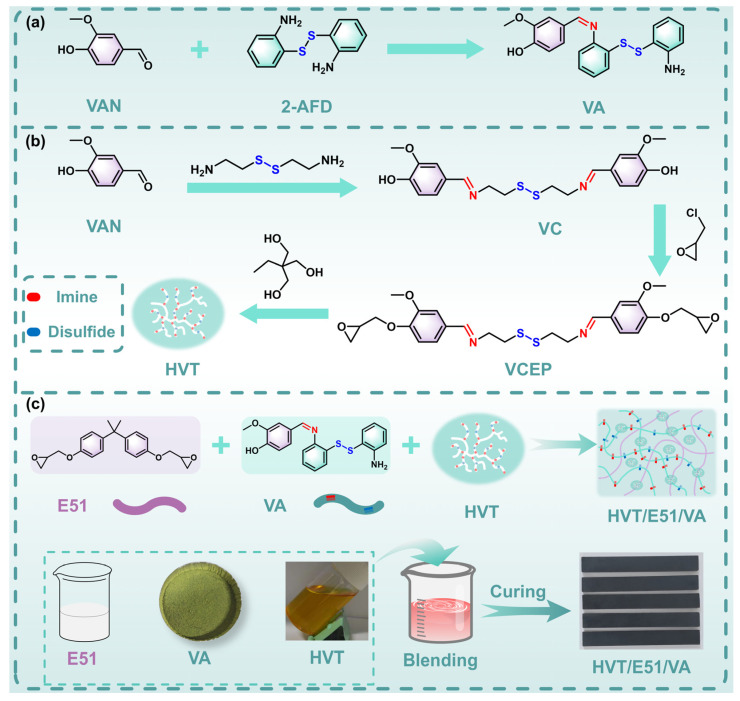
Synthesis of (**a**) VA, and (**b**) HVT. (**c**) Schematic diagram for the preparation of HVT/E51/VA.

**Figure 2:**
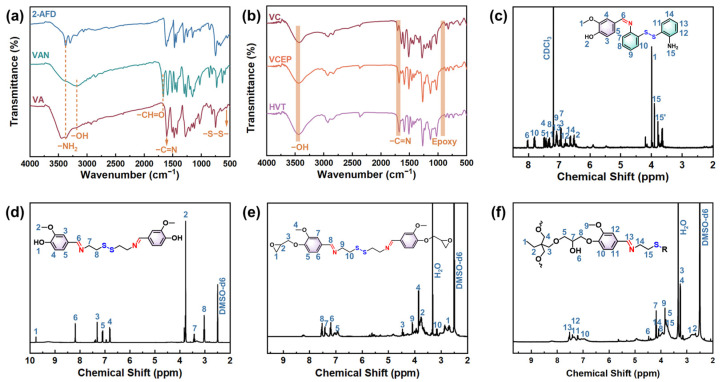
FT-IR spectra of (**a**) 2-AFD, VAN, and VA, (**b**) VC, VCEP, and HVT; ^1^H NMR spectra of (**c**) VA, (**d**) VC, (**e**) VCEP, and (**f**) HVT (where R represents the other half of the shown fragment).

As shown in Figure 1a, 2,2′-dithiodianiline (2-AFD, 1 mol) was added into a four-necked flask with ethanol (EtOH) as the solvent. Vanillin (VAN, 1 mol) was then added dropwise and reacted at 60 °C for 4 h under mechanical agitation. The vanillin-based curing agent (VA) was finally obtained after precipitation with petroleum ether and drying at 80 °C for 12 h (yield: 89.5%).

### 2.3. Synthesis of Vanillin-Based Hyperbranched Epoxy Toughening Agent (HVT)

Firstly, cystamine dihydrochloride (Cys-HCl, 0.3 mol) was reacted with NaOH aqueous solution (0.63 mol) at room temperature for 3 h to obtain cystamine (Cys). Then, VAN (0.6 mol) was further reacted with Cys at 60 °C for 4 h by using ethanol as the solvent. After precipitation with petroleum ether and drying at 80 °C for 12 h, the intermediate VC was obtained (yield: 92.1%).

VC (0.3 mol) and epichlorohydrin (ECH, 3.1 mol) were first reacted at 90 °C for 3 h by using benzyltriethylammonium chloride as the catalyst. Then, 40 wt% NaOH solution (120 mL) was added dropwise, and reacted at 30 °C for 4 h. VCEP was obtained after filtration and removal of the solvent via rotary evaporation (yield: 78.4%). After further reaction with TMP (0.2 mol) in dimethyl sulfoxide at 120 °C for 4 h, the hyperbranched epoxy toughening agent (HVT) was obtained (yield: 75.8%). The synthetic route is displayed in Figure 1b.

### 2.4. Curing of Epoxy Resin

As depicted in Figure 1c, E51, VA, and HVT were mixed together at 70 °C for 30 min. After vacuum degassing, the homogeneous mixture was poured into a steel mold and cured at 90 °C × 3 h + 135 °C × 3 h + 185 °C × 3 h. The curing program was analyzed by DSC, and the details are provided in Section S4. The molar ratio of E51 to VA is 10:7. The cured resin is named xHVT/E51/VA, where x denotes the mass ratio of HVT to E51.

### 2.5. Determination of Gel Content

The gel content was determined by the Soxhlet extraction method. Approximately 1.0 g of the cured resin (m_0_) was extracted with acetone under reflux for 48 h. The residue was then dried in a vacuum oven at 80 °C to a constant weight (m_1_). The gel content was calculated according to the following formula:

(1) $$\mathrm{Gel}\;\mathrm{content}=\frac{m_{1}}{m_{0}}\times 100\%$$

### 2.6. Reprocessing of the HVT/E51/VA

The broken HVT/E51/VA was first ground into powder, sieved through an 80-mesh screen, then underwent hot pressing at 150 °C under 10 MPa for 4 h. The obtained reprocessing samples are named Rx-5HVT/E51/VA, where x represents reprocessing cycles.

### 2.7. Degradation of the HVT/E51/VA

The degradation of HVT/E51/VA was assessed by immersing a 0.20 g sample in the solvent at 80 °C while recording the time for complete degradation. The degradation rate (ν_d_, mg·mL^−1^·h^−1^) is calculated according to the following equation:

(2) $$\nu_{d}=\frac{m_{1}-m_{0}}{V\cdot t}$$

where m_0_ and m_1_ represent the mass of the resin before and after degradation, respectively; t is the time of complete degradation, and V is the volume of the solvent.

After complete degradation, the solvent was evaporated using a rotary evaporator. The resulting substance was further analyzed using a Fourier infrared spectrometer (Nicolet 5700, Thermo Fisher, Waltham, MA, USA).

### 2.8. Self-Healing of the HVT/E51/VA

The HVT/E51/VA was first scratched with a knife, then heated at 180 °C for 4 h without external pressure. The self-healing performance of the sample was evaluated by observing the depth and width of scratches via a laser confocal 3D microscope (VK-X100K, Keyence, Osaka, Japan).

### 2.9. Characterization

The chemical structures of VA, VC, VCEP, and HVT were analyzed by a Fourier infrared spectrometer (Nicolet 5700, Thermo Fisher, Waltham, MA, USA). ^1^H NMR spectra were obtained on a ^1^H NMR spectrometer at 400 MHz (Avance III, Bruker, Bremen, Germany) using CDCl_3_ or DMSO-d6 as the solvent and tetramethylsilane as the internal reference. The molecular weight of HVT was measured by GPC (Waters 2414, Waters, Milford, MA, USA) using THF as the mobile phase at a flow rate of 1 mL·min^−1^ and 40 °C. Polystyrene (PS) standards were used for calibration. DMA was performed on a dynamic mechanical analyzer (DMA 1, METTLER TOLEDO, Greifensee, Switzerland) in single three-point bending mode at 1 Hz with a heating rate of 3 °C·min^−1^ from 50 to 250 °C. Bending and impact strength were measured by an electronic universal testing machine (CMT4024, SANS, Shenzhen, China) and a cantilever beam impact testing machine (CEAST 950, CEAST, Pianezza, Italy) with the sample size of 80 × 10 × 4 mm^3^, respectively. The surface morphology was observed on a laser confocal 3D microscope (VK-X100K, Keyence, Osaka, Japan) and a scanning electron microscope (S-3400N, Hitachi, Tokyo, Japan), respectively. The thermal stability was evaluated by a simultaneous thermal analyzer (STA449C, NETZSCH, Selb, Germany) at a heating rate of 10 °C·min^−1^ in N_2_.

## 3. Results and Discussion

### 3.1. Chemical Structure of VA and HVT

As shown in the FT-IR spectrum of VA depicted in Figure 2a, the stretching vibration peaks of the –CH=O are absent at 1667 cm^−1^, and the stretching vibration peaks of the –NH_2_ at 3554 cm^−1^ are weakened compared to those of 2-AFD. Meanwhile, the stretching vibration peaks of the −C=N are observed at 1609 cm^−1^. This confirms that the −CH=O of VAN reacts with part of the −NH_2_ of 2-AFD via an aldehyde–amine condensation reaction to form imine bonds. Furthermore, the persistence of the −OH absorption peaks near 3160 cm^−1^ in the spectrum of VA indicates that the −OH does not react with the −NH_2_. In addition, the characteristic peak of the disulfide bond belonging to 2-AFD appears at 562 cm^−1^ in the FT-IR spectrum of VA, demonstrating that the disulfide bond is successfully retained and introduced into VA. These results preliminarily indicate the successful synthesis of VA containing both imine and disulfide bonds. The ^1^H NMR spectrum of VA is presented in Figure 2c, and detailed data are provided in Section S1. The peak at 8.03 ppm is attributed to the imine proton, and the signals at 3.85 and 3.68 ppm correspond to the two hydrogen atoms of the –NH_2_. The integration area ratio of the –NH_2_ protons to the imine proton is 2:1, which is consistent with the mono-substituted structure. The peaks in the range of 6.6–7.8 ppm are assigned to the aromatic ring protons, the peak at 6.5 ppm is assigned to the phenolic hydroxyl proton, and the peak at 3.99 ppm corresponds to the methoxy protons. It also confirms the successful synthesis of VA.

The FT-IR spectra of VC, VCEP, and HVT are shown in Figure 2b. For VC, the emergence of the imine (–C=N) stretching vibration peaks at 1632 cm^−1^, along with the disappearance of characteristic peaks for –NH_2_ and –CHO, confirms the complete reaction of Cys with VAN. The epoxy peak is observed at 915 cm^−1^ in the spectrum of VCEP. Compared to the VCEP, the −OH peak of the HVT at 3343 cm^−1^ is enhanced, while the epoxy group peak at 911 cm^−1^ is weakened. This is due to the reaction between the epoxy group of VCEP and the hydroxyl group of TMP.

The ^1^H NMR spectra are displayed in Figure 2d–f. For VC, the −CH=N peak appears at 8.2 ppm. The resonances at 4.4 ppm, 3.6 ppm, and 2.7 ppm in Figure 2e are attributed to the protons on the epoxy group of VCEP. In Figure 2f, the peaks at 3.7 ppm, 3.2 ppm, and 3.1 ppm are assigned to the −O−CH_2_, and the peak at 4.7 ppm is attributed to the −OH. It also confirms that HVT has been successfully synthesized. Detailed data are provided in Section S1.

The degree of branching (DB) of HVT, calculated according to the ^1^H NMR spectrum in Figure S3, is 0.54, suggesting its highly branched structure. The calculation details are provided in Section S2, and the GPC curve of HVT is shown in Figure S4. The number-average molecular weight (M_n_), weight-average molecular weight (M_w_), and PDI of HVT are 1500 g·mol^−1^, 2500 g·mol^−1^, and 1.67, respectively.

### 3.2. Curing Behavior of the HVT/E51/VA

**Figure 3:**
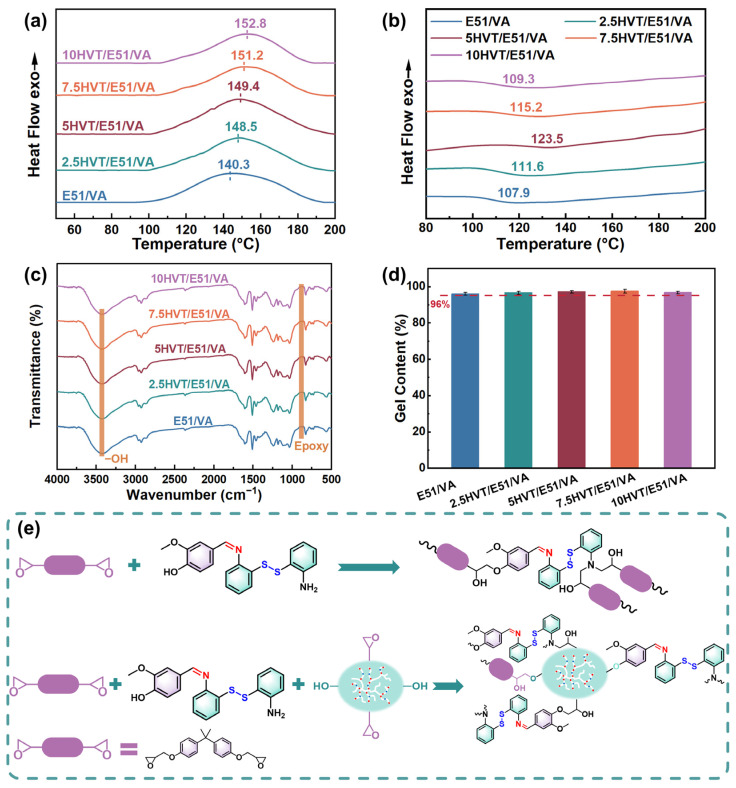
DSC curves of E51/VA and HVT/E51/VA (**a**) before and (**b**) after curing reaction; (**c**) FT-IR spectra, (**d**) the gel contents, and (**e**) curing mechanism of E51/VA and HVT/E51/VA.

The curing behavior of E51/VA with different contents of HVT was investigated by DSC. All curves show a single exothermic peak (Figure 3a), suggesting good compatibility and homogeneous reaction between HVT and E51/VA. In addition, as the HVT content is increased, the curing peak temperature is also slightly raised. The steric hindrance arising from the rigid benzene rings and the hyperbranched topological structure in HVT may impede the nucleophilic attack of the amino groups on the epoxy groups [36,37]. Furthermore, the introduction of HVT increases the viscosity of the curing system and reduces the mobility of chain segments, thereby inhibiting the diffusion and collision of reactive groups. For all cured samples, no exothermic peak is observed in the range of 50–200 °C (Figure 3b), indicating complete curing of E51/VA and HVT/E51/VA. The FT-IR spectra of the cured HVT/E51/VA are depicted in Figure 3c. Both the −NH_2_ characteristic peak at 3500 cm^−1^ and the epoxy peak at 915 cm^−1^ disappear after the curing reaction. The gel contents of both the E51/VA and HVT/E51/VA systems are above 96%, further confirming complete curing of the resins and the formation of a highly cross-linked network (Figure 3d). As the curing mechanism illustrated in Figure 3e, the amine in VA first reacted with epoxy groups through nucleophilic addition at lower temperatures due to their stronger nucleophilic activity. After the –NH_2_ groups were fully consumed, the hydroxyl groups of VA and HVT further etherified with the residual epoxy groups at high temperature, forming a dense three-dimensional network.

### 3.3. Dynamic Thermo-Mechanical Properties of the HVT/E51/VA

**Figure 4:**
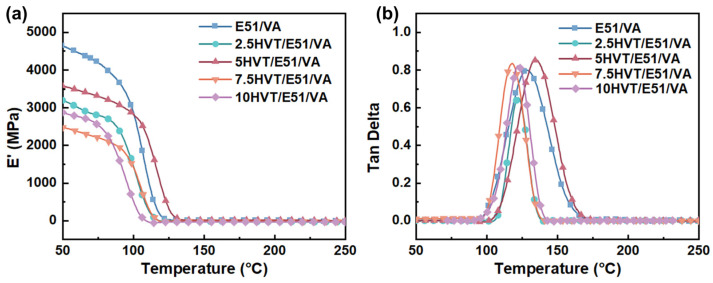
(**a**) Storage modulus and (**b**) loss factor of E51/VA and HVT/E51/VA.

As shown in Figure 4a, the storage modulus of the epoxy resin decreases with the addition of HVT. The hyperbranched topological structure of HVT forms intramolecular cavities, which reduces the packing density of the polymer chains, thereby leading to a decrease in the storage modulus. In addition, the *T*_g_ of the E51/VA, determined by the peak temperature of the loss factor (tan δ), is 128.6 °C. Notably, when the addition of HVT is 5 wt%, the *T*_g_ of the resin is raised to 134.7 °C. The multifunctional terminal groups of HVT can participate in the curing reaction, which increases the crosslinking density of the system and restricts the motion of molecular chains, thereby increasing the *T*_g_. Meanwhile, in Figure 4b, all tan δ curves of the HVT/E51/VA exhibit a sharp single peak, indicating homogeneity of the system and good compatibility between the HVT and E51.

The variation in the cross-link density (ν_e_) of E51/VA with HVT content, calculated according to Equation (3), is shown in Table 1 [36,38,39].

(3) $$\nu_{e}=\frac{E^{\prime }}{3RT}$$

where E′ is the storage modulus at the temperature of *T*_g_ + 30 °C, R represents the gas constant, and *T* is the Kelvin temperature.

The cross-link density of E51/VA is 2044 mol·m^−3^. When the HVT content is 2.5 wt%, the highly branched HVT prefers to form a contracted spherical conformation. Some entrapped reactive terminal groups may be unable to participate in the curing reaction, leading to a low cross-link density of 1554 mol·m^−3^. As the content of HVT is raised to 5 wt%, however, additional chemical crosslinking points and physical entanglement sites are introduced into the cross-linked network by the hyperbranched topological structure and multifunctional terminal groups of HVT. Thus, the cross-link density of the system is enhanced to 2561 mol·m^−3^. The steric hindrance is also enhanced with further increases in HVT content. Hence, the cross-link densities of the resins with 7.5 wt% and 10 wt% HVT are slightly reduced, but they are still slightly higher than that of E51/VA.

### 3.4. Thermal Stability of the HVT/E51/VA

**Figure 5:**
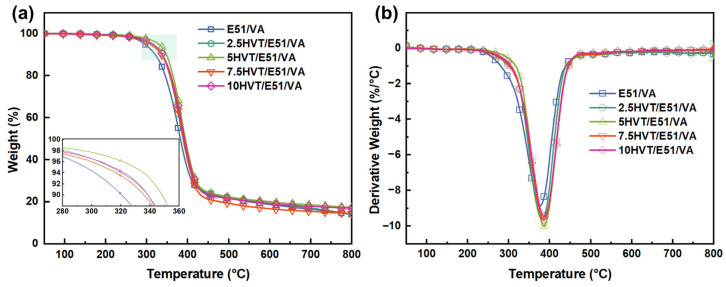
(**a**) TGA and (**b**) DTG curves of E51/VA and HVT/E51/VA.

As illustrated in Figure 5a, the 5% weight loss decomposition temperature (*T*_d5%_) of E51/VA is 295.6 °C. The imine and disulfide bonds have relatively low dissociation energies and therefore prefer to cleave at high temperatures [38,40]. However, the thermal stability is significantly enhanced after the introduction of HVT. Particularly for 5HVT/E51/VA, its *T*_d5%_ is raised to 329.1 °C, and its maximum decomposition temperature (*T*_max_) to 379.7 °C as displayed in Figure 5b. The abundant aromatic rings in HVT can suppress the cleavage of chemical bonds in the early stage of thermal decomposition. Meanwhile, the cross-link density of the system is raised with the introduction of HVT, forming a more stable three-dimensional network. As a result, the thermal stability of E51/VA is greatly enhanced with the addition of HVT.

To further compare thermal stability of E51/VA and HVT/E51/VA, the thermal stability index (*T*_s_) was calculated according to Equation (4), and the results are listed in Table 2.

(4) $$T_{s}=0.49\left[T_{d5\%}+0.6\left(T_{d30\%}-T_{d5\%}\right)\right]$$

It is observed that *T*_s_ of the systems incorporating HVT is much higher than that of E51/VA. When the HVT content is 5 wt%, *T*_s_ reaches 174.8 °C, 11.1 °C higher than the unmodified resin.

### 3.5. Mechanical Properties of the HVT/E51/VA

**Figure 6:**
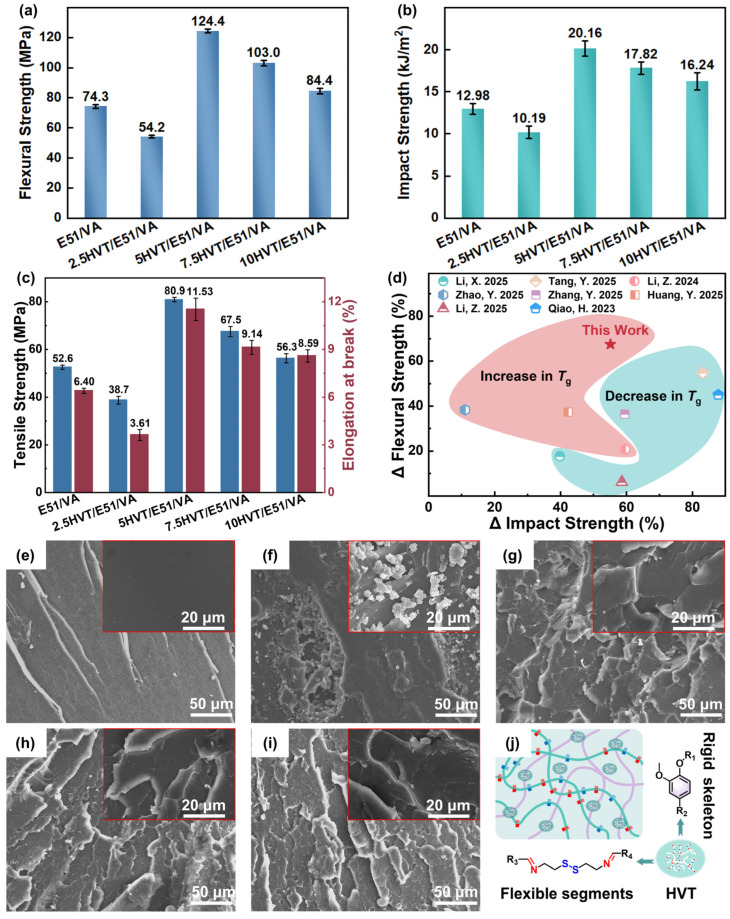
(**a**) Flexural strength, (**b**) impact strength, and (**c**) tensile strength and elongation at break of E51/VA and HVT/E51/VA; (**d**) comparison with properties from the previous literature [26,30,36,38,39,41,42,43]; SEM images of fracture surfaces of (**e**) E51/VA, (**f**) 2.5HVT/E51/VA, (**g**) 5HVT/E51/VA, (**h**) 7.5HVT/E51/VA, and (**i**) 10HVT/E51/VA; (**j**) the toughening and reinforcing mechanism of HVT.

The mechanical properties of HVT/E51/VA were further evaluated, and the variation in flexural and impact strength with HVT content is shown in Figure 6a,b. The stiff benzene rings in the structure and the highly cross-linked network lead to the inherent brittle nature of epoxy. As a result, E51/VA shows relatively low mechanical strength.

When 2.5 wt% HVT is added to the system, both flexural and impact strength are reduced. This may be ascribed to the highly branched architecture of HVT, which tends to form micro-spherical structures at low concentrations, thus introducing some defects into the system [38]. As the HVT concentration is further increased, mechanical properties are enhanced accordingly. The flexural and impact strength of the resins with 5 wt% HVT reach a maximum of 124.4 MPa and 20.16 kJ/m^2^, which are improved by 71.63% and 55.32% compared to E51/VA, respectively. The highly branched structure of HVT efficiently dissipates energy under external stress, thereby imparting excellent toughness to the epoxy resins. Beyond this optimum concentration, their strengths gradually decrease, but are still higher than those of E51/VA. This is due to the introduction of more aliphatic segments from HVT, as well as the reduced cross-link density. Tensile properties of E51/VA and HVT/E51/VA were further compared, and the results are shown in Figure 6c. The trends of tensile strength and elongation at break are similar to those of flexural and impact strength. When the HVT content is 5 wt%, the tensile strength reaches its maximum value of 80.9 MPa and the elongation at break is 11.53%, which are 53.80% and 80.16% higher than those of unmodified E51/VA, respectively. In addition, HVT exhibits superior reinforcing, a toughening effect, and a synergistic increase in thermal properties when compared to the other hyperbranched epoxy toughening agents in the literature [26,30,36,38,39,41,42,43], as shown in Figure 6d.

To further elucidate the influence of HVT on the mechanical properties of E51/VA, the morphologies of the impact fracture surfaces of the samples were observed, and the images are displayed in Figure 6e–i. The E51/VA exhibits a fairly smooth fracture surface with some minor and ordered cracks in Figure 6e. This is typical of brittle fracture, indicating a low capacity for energy dissipation when subjected to external force. In Figure 6f, there are numerous micro-spheres on the fracture surface of 2.5HVT/E51/VA. They may act as stress points, resulting in a decrease in strength and toughness of the resins [44]. So, its flexural and impact strength are the lowest among all samples. On the contrary, the fracture surface of 5HVT/E51/VA (Figure 6g) is much rougher, characterized by prominent crack deflection and substantial plastic deformation, thus effectively promoting energy absorption and dissipation. As illustrated in Figure 6j, the HVT molecule is composed of a rigid aromatic skeleton and flexible aliphatic segments. When cracks occur, the rigid aromatic skeletons facilitate crack deflection, thereby significantly prolonging the crack propagation path. Concurrently, the flexible segments can induce the formation and expansion of shear bands in stress-concentration zones, thus greatly promoting energy absorption and dissipation. Furthermore, the intramolecular cavities formed by hyperbranched HVT increase the local free volume of the epoxy resin, resulting in effective energy dissipation [45]. Therefore, both toughness and strength are enhanced simultaneously. However, as the content of HVT is further increased to 7.5 wt% and 10 wt%, the surfaces become relatively smooth again, as displayed in Figure 6h,i. The aggregation of HVT leads to localized stress concentration. The resins exhibit low plastic deformation resistance. The results of surface morphologies agree well with those of flexural and impact strength of the resins.

### 3.6. Reprocessing Performance of the HVT/E51/VA

**Figure 7:**
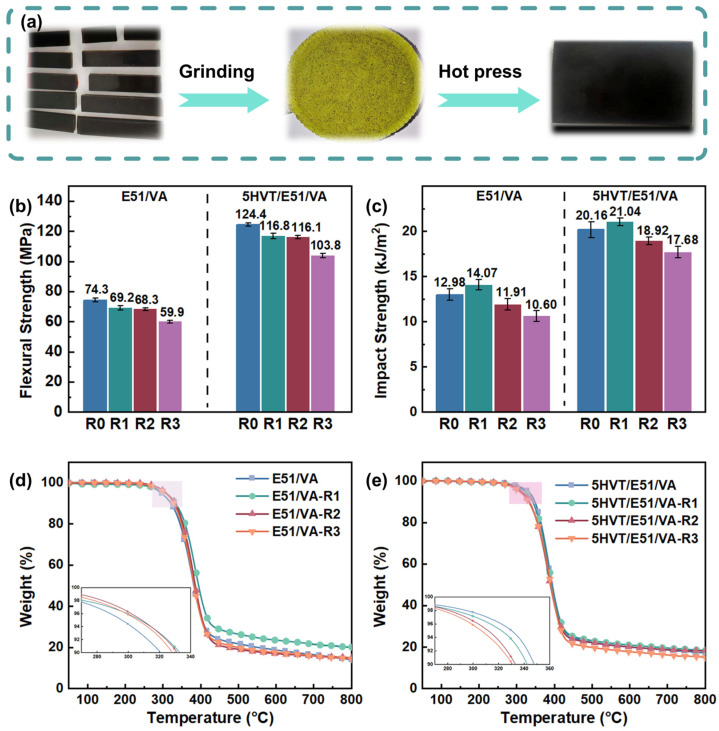
(**a**) Schematic diagram of the reprocessing process; (**b**) flexural strength, and (**c**) impact strength of E51/VA and 5HVT/E51/VA before and after reprocessing; TGA curves of (**d**) E51/VA-R and (**e**) 5HVT/E51/VA-R.

As illustrated in Figure 7a, all samples prepared with VA as the curing agent can be reprocessed by the hot-pressing method. The flexural and impact strength of the resins before and after reprocessing are compared in Figure 7b,c. It can be seen that both E51/VA and 5HVT/E51/VA exhibit remarkably high strength retention rates, owing to dual dynamic bonds in the network. Especially, for 5HVT/E51/VA-R1, 5HVT/E51/VA-R2 and 5HVT/E51/VA-R3, the flexural strength retention rates reached 93.89%, 93.33%, and 83.44%, respectively. The impact strength retention rates show a similar tendency to change. It is even raised to 108.37% for E51/VA-R1, and 104.36% for 5HVT/E51/VA-R1. The dynamic exchange reactions of imine and disulfide bonds promote topological rearrangement of the cross-linked network, which may eliminate internal stress and local defects generated during the initial curing process. This helps to absorb and dissipate impact energy more effectively. In addition, a small amount of unreacted epoxy, hydroxyl, or amino groups can further react at high temperature during reprocessing, leading to a slight enhancement in cross-link density. Consequently, the impact strength is raised slightly after the first reprocessing cycle. Nevertheless, the partial irreversible damage to the dynamic bonds at repeated high temperature eventually leads to a subsequent decline in performance of the resins.

In addition, as the results of paired sample *t*-test shown in Table S2, both the flexural and impact strengths of 5HVT/E51/VA-R are much higher than those of E51/VA-R, with a mean difference of 47.35 MPa and 7.06 kJ/m^2^, respectively. It demonstrates that the addition of 5 wt% HVT can obviously improve the mechanical properties of 5HVT/E51/VA, and that those improvements are retained after reprocessing.

The thermal stability of recycled samples after reprocessing was further evaluated by TGA. The curves are displayed in Figure 7d,e, and the relevant data are listed in Table 3. Both E51/VA-R and 5HVT/E51/VA-R exhibit excellent thermal stability. After reprocessing, the *T*_d5%_ of E51/VA is raised from 295.6 to 306.5 °C. This should be attributed to the formation of a denser cross-linked network during hot pressing. The *T*_d5%_ of the 5HVT/E51/VA-R is slightly decreased after three reprocessing cycles, due to the partial scission of flexible segments in HVT under repeated thermo-mechanical load; however, it still remains at 305.7 °C.

### 3.7. Degradation Performance of the HVT/E51/VA

**Figure 8:**
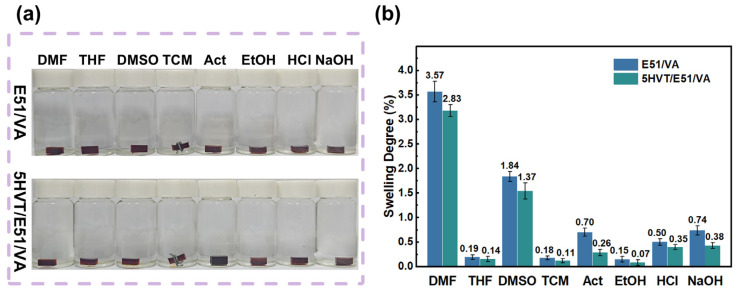
(**a**) Photographs after solvent immersion and (**b**) swelling degree of E51/VA and 5HVT/E51/VA.

As shown in Figure 8a, negligible changes are observed for both E51/VA and 5HVT/E51/VA even after 48 h of immersion in the solvents, such as tetrahydrofuran (THF), dimethylformamide (DMF), trichloromethane (TCM), dimethyl sulfoxide (DMSO), acetone (Act), ethanol (EtOH), 1 M HCl, and 1 M NaOH. The mass loss rates of all samples after immersion are less than 0.1% (Table S2). Furthermore, the swelling degrees of E51/VA and 5HVT/E51/VA are both lower than 1% in most solvents except for DMSO and DMF, as shown in Figure 8b. The latter is much lower for the higher cross-link density of 5HVT/E51/VA. They exhibit excellent chemical stability and solvent resistance.

**Figure 9:**
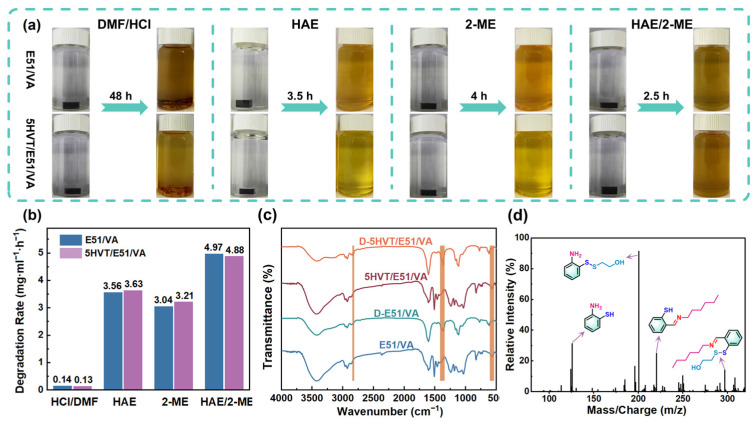
(**a**) Degradation process in various solvents and (**b**) degradation rates of E51/VA and 5HVT/E51/VA, (**c**) FT-IR spectra, and (**d**) TOF-MS spectra of degradation products.

However, as presented in Figure 9a, when E51/VA and 5HVT/E51/VA were immersed in HCl/DMF (3:7, *v*/*v*) at 80 °C for 24 h, the samples began to degrade, accompanied with the color of solution turning yellow. After 48 h, some undegraded fragments can still be observed. The mass of E51/VA and 5HVT/E51/VA after immersion in HCl/DMF for 48 h decreased by 51.21% and 49.06%, respectively. The imine bond can hydrolyze into ammonium salt and aldehyde under acidic conditions, while the highly cross-linked network of the epoxy resins prevents H^+^ ions from penetrating and diffusing, resulting in low degradation efficiency. In contrast, when the samples were immersed in hexylamine (HAE) or 2-mercaptoethanol (2-ME) at 80 °C, a complete degradation was achieved in 3.5 or 4 h, respectively. Furthermore, E51/VA and 5HVT/E51/VA can fully degrade in a mixed solvent of HAE and 2-ME (5:5, *v*/*v*) within only 2.5 h at 80 °C. As shown in Figure 9b, the degradation rates of E51/VA and HVT/E51/VA show little difference in various solvents, suggesting that the incorporation of HVT has no significant effect on resin degradation behavior. In addition, both systems degrade rather slowly in HCl/DMF, but more rapidly in HAE, 2-ME, and HAE/2-ME. Particularly in the HAE/2-ME, the degradation rates of E51/VA and HVT/E51/VA reach 4.97 and 4.88 mg·mL^−1^·h^−1^, respectively.

Further FT-IR analysis of the degradation products is depicted in Figure 9c. In the FT-IR spectra of the degradation products of E51/VA and 5HVT/E51/VA, the characteristic peaks of disulfide bonds at 564 cm^−1^ disappear. Meanwhile, new characteristic peaks appear at 2828 cm^−1^, assigned to the stretching vibration of the thiol group (−SH). Furthermore, no characteristic peaks of the imine bonds at 1622 cm^−1^ are observed in the degradation products. At the same time, the stretching and bending vibrational peaks of the primary amine group appear at 3151 cm^−1^ and 1384 cm^−1^, respectively. These directly confirm the occurrence of the thiol–disulfide exchange reaction and the imine exchange reaction. In addition, the degradation products of 5HVT/E51/VA were further analyzed by TOF-MS, and the results are shown in Figure 9d. The degradation products mainly consisted of the four small molecules, indicating great collapse of the cross-linked network.

**Figure 10:**
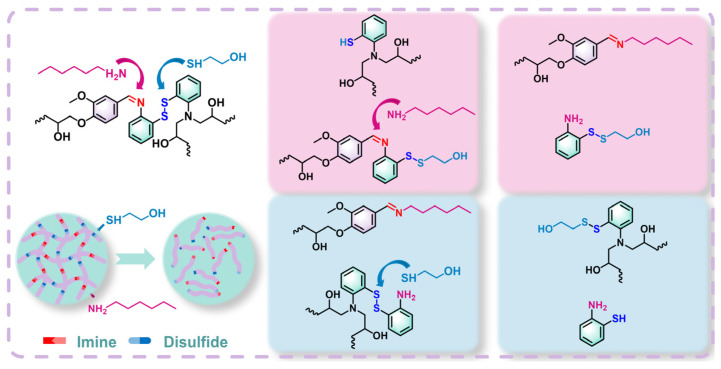
Degradation mechanism of E51/VA and 5HVT/E51/VA.

As schematically illustrated in Figure 10, HAE primarily attacks bonds via the imine exchange reaction, while 2-ME cleaves bonds through thiol–disulfide exchange. Both imine and disulfide dynamic bonds are broken simultaneously in the HAE/2-ME mixed solvent, leading to a much faster depolymerization of the cross-linked network from two different sites. In addition, once the dense three-dimensional network collapses, the penetration and attack of the reagents become easier.

### 3.8. Self-Healing Performance of the HVT/E51/VA

**Figure 11:**
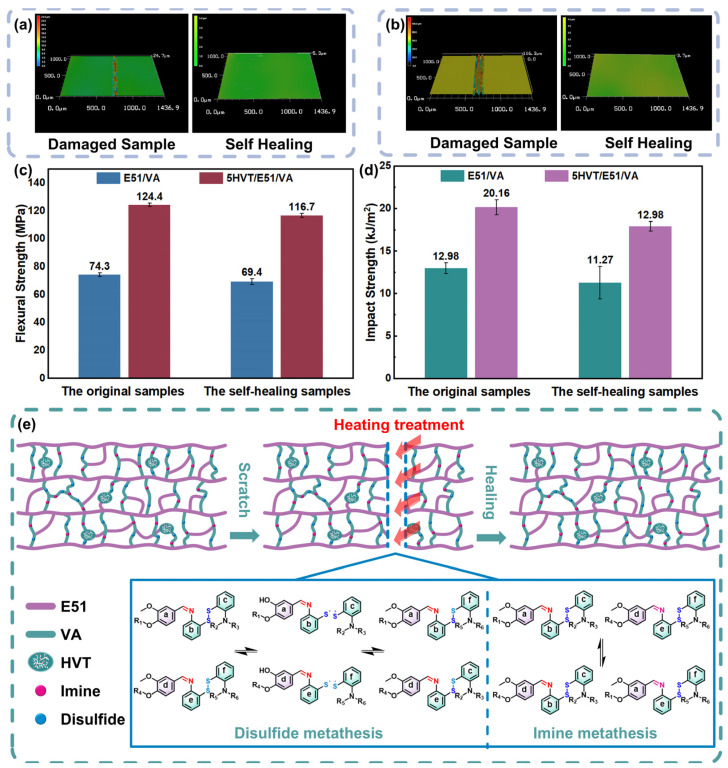
Self-healing performance of (**a**) E51/VA and (**b**) 5HVT/E51/VA, (**c**) flexural and (**d**) impact strengths before and after self-healing of E51/VA and 5HVT/E51/VA, (**e**) self-healing mechanism of HVT/E51/VA.

It can be seen that the scratches of the E51/VA and 5HVT/E51/VA completely disappeared after 4 h of heating, as displayed in Figure 11a,b. In addition, the flexural and impact strengths of the self-healed samples were measured, and the results are shown in Figure 11c,d. The healing efficiencies of flexural and impact strengths for E51/VA are 93.4% and 86.8%, respectively, while those of 5HVT/E51/VA increased to 93.8% and 88.9%, respectively. Both resins possess excellent self-healing ability, owing to the dynamic exchange reactions of disulfide and imine bonds in the network.

Typically, the segmental motion could be activated when heated above *T*_g_ of the polymers [11]. It promotes the contact of fractured surfaces and the exchange reaction of disulfide and imine bonds, resulting in scratch self-healing. As presented in Figure 11e, the imine bonds reversibly cleave and recombine with neighboring imine bonds upon heating, leading to rearrangement of the covalent network [46]. Simultaneously, the thiyl radicals generated by disulfide bonds under thermal energy would attack other disulfide bonds to reorganize the network. Under the synergistic action of these two dynamic bonds, the broken covalent networks are rebuilt, resulting in an efficient self-healing performance.

## 4. Conclusions

The curing agent (VA) and hyperbranched epoxy toughening agent (HVT) containing imine and disulfide dual dynamic covalent bonds were synthesized from vanillin. Benefiting from the unique hyperbranched topology and abundant terminal epoxy groups of HVT, the improved E51 epoxy resin exhibits significantly enhanced mechanical properties. When the concentration of HVT is 5 wt%, the impact and flexural strengths of the cured resins are improved by 55.32% and 71.63%, respectively, compared to E51/VA. The thermal stability of the 5HVT/E51/VA is also enhanced. Furthermore, 5HVT/E51/VA demonstrates excellent reprocessing and self-healing ability. Even after three reprocessing cycles, the flexural and impact strength retention rates of the resins exceed 80%. Moreover, the material shows superior chemical and solvent resistance at room temperature, but completely degrades in hexylamine/2-mercaptoethanol mixed solvents within 2.5 h. This research provides a sustainable method for high-performance epoxy resins, suggesting their potential applications in self-healing and reprocessable composites.

## Figures and Tables

**Table 1:** The cross-link density of E51/VA and HVT/E51/VA.

Sample	*T*_g_ (°C)	E′ (MPa)	ν_e_ (mol·m^−3^)
E51/VA	128.6	20.48	2044
2.5HVT/E51/VA	121.5	15.30	1554
5HVT/E51/VA	134.7	26.64	2561
7.5HVT/E51/VA	122.3	21.31	2160
10HVT/E51/VA	117.2	20.04	2058

**Table 2:** Thermal stability of E51/VA and HVT/E51/VA.

Sample	*T*_d5%_ (°C)	*T*_d30%_ (°C)	*T*_s_ (°C)
E51/VA	295.6	359.9	163.7
2.5HVT/E51/VA	314.2	368.4	169.9
5HVT/E51/VA	329.1	375.2	174.8
7.5HVT/E51/VA	308.7	369.9	169.3
10HVT/E51/VA	314.6	371.5	170.9

**Table 3:** Thermal stability of E51/VA and HVT/E51/VA before and after reprocessing.

Sample	*T*_d5%_ (°C)	*T*_d30%_ (°C)	*T*_s_ (°C)
E51/VA	295.6	359.9	163.7
E51/VA-R1	306.5	372.6	169.6
E51/VA-R2	307.6	366.0	167.9
E51/VA-R3	304.8	362.2	166.2
5HVT/E51/VA	329.1	375.2	174.8
5HVT/E51/VA-R1	321.3	373.3	172.7
5HVT/E51/VA-R2	310.9	368.3	169.2
5HVT/E51/VA-R3	305.7	369.5	168.6

## Data Availability

The original contributions presented in this study are included in the article/Supplementary Materials. Further inquiries can be directed to the corresponding author.

## Supplementary Materials

The following supporting information can be downloaded at: https://www.mdpi.com/article/10.3390/polym18121418/s1, Figure S1: The chemical formula of VA, VC, VCEP, and HVT; Figure S2: The dendritic, terminal, and linear units of HVT; Figure S3: Partial ^1^H NMR spectrum of HVT, (a) –CH_3_, (b) –OH; Figure S4: GPC curve of HVT; Figure S5: (a) DSC curves at different heating rates; the fitting curves for 5HVT/E51/VA based on (b) Kissinger and (c) Ozawa equations; (d) extrapolation curves of T-β; Table S1: Molecular weight and distribution of HVT; Table S2. Mass of E51/VA and 5HVT/E51/VA before and after immersion in different solvents; Table S3: Mechanical properties and paired *t*-test of E51/VA-R and 5HVT/E51/VA-R.
