# Supplementary material for: Toughening, Reinforcing, and Reprocessing of Epoxy Resin with Hyperbranched Polymer Containing Disulfide and Imine Dual Dynamic Covalent Bonds
Source: Polymers (Basel). 2026 Jun 6;18(12):1418. doi: 10.3390/polym18121418 (PMC13306418; doi:10.3390/polym18121418)
Supplement: Supplementary file 1 [file polymers-18-01418-s001.zip › polymers-4325407-supplementary.pdf]

polymers

MDPI

Supplementary Materials

Toughening, reinforcing, and reprocessing of Epoxy Resin with Hyperbranched Polymer containing disulfide and imine dual dynamic covalent bonds

Xu Sun , Chen He and Yan Zhang *

Key Laboratory of Specially Functional Polymeric Materials and Related Technology (Ministry of Education), School of Materials Science and Engineering, East China University of Science and Technology, Shanghai 200237, China; y30230778@mail.ecust.edu.cn (X.S.); y82240304@mail.ecust.edu.cn (C.H.)

* Correspondence: yzhang@ecust.edu.cn

S1. ^{1}H-NMR assignments of VA, VC, VCEP, and HVT

^{1}H NMR (400 MHz, $CDCl_3$) of VA: δ 8.03 (1H, 6), 7.81 (1H, 10), 7.49 (1H,4), 7.46 – 7.40 (1H, 5), 7.35 (1H, 8), 7.10 (1H, 11), 7.08 (1H, 9), 7.08 – 7.06 (1H, 13), 6.99 – 6.95 (1H, 7), 6.93 (1H, 3), 6.85 – 6.79 (1H, 12), 6.66 – 6.63 (1H, 14), 6.50 (1H,2), 3.99 (3H, 1), 3.85 (2H, 15).

^{1}H NMR (400 MHz, DMSO-d6) of VC: δ 9.76 (1H, 1), 8.19 (1H, 6), 7.31 (1H, 3), 7.10 (1H, 5), 6.81 (1H, 4), 3.78 (3H,7), 3.77 (2H, 2), 3.03 (2H, 8).

^{1}H NMR (400 MHz, DMSO-d6) of VCEP: δ 9.85 (1H, 8), 7.58 – 7.49 (1H, 7), 7.44 – 7.33 (1H, 6), 6.93 (1H, 5), 4.95 – 4.36 (2H, 3), 4.13 – 3.97 (2H, 9), 3.85 (3H, 4), 3.77 (1H, 2), 3.24 – 2.96 (2H, 10), 2.88 – 2.80 (2H, 1).

^{1}H NMR (400 MHz, DMSO-d6) of HVT: δ 7.53 (1H, 13), 7.41 (1H, 11), 7.34 (1H, 12), 7.20 (1H, 10), 4.51 – 4.32 (1H, 6), 4.18 (2H, 7), 4.08 (2H, 14), 3.93 (2H, 18), 3.84 (3H, 9), 3.81 (2H, 5), 3.74 (2H, 15), 3.25 (2H, 3), 3.24 (2H, 4), 2.75 (3H, 1), 2.69 (2H, 2).

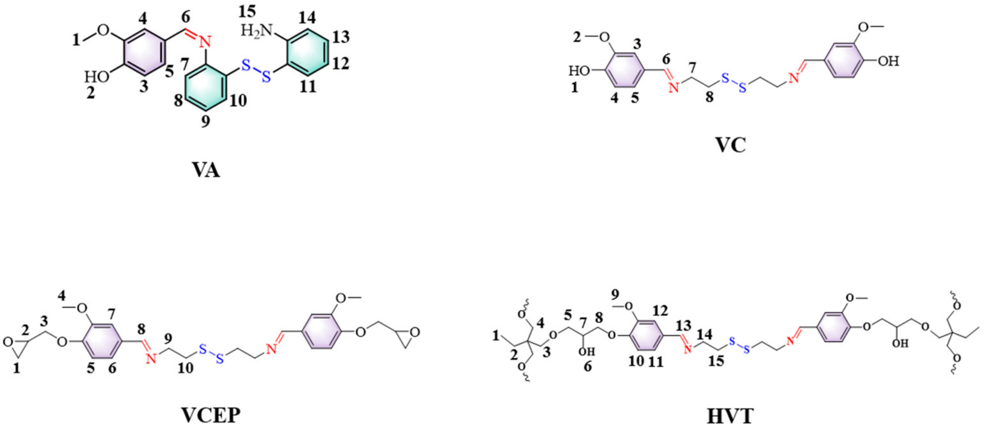

Figure S1. The chemical formula of VA, VC, VCEP, and HVT.

S2. The degree of branching of HVT

The degree of branching (DB) refers to the molar ratio of dendritic units and terminal units to the total structural units. A higher DB value indicates a more compact molecular structure and greater internal cavity volume. DB can be calculated using Eq. S1.

$$DB = \frac{D + T}{D + L + T} \quad \text{(S1)}$$

Where D is the dendritic unit; T is the terminal unit; L is the linear unit. Their relative ratios were determined by ^{1}H NMR integration (Figure S2), and their chemical structures are shown in Figure S2.

The peak at 3.13 ppm corresponds to the hydroxyl proton (-OH) of the L units in Figure S3(a). Therefore, its intensity value represents the number of L units. As shown in Figure S3(b), the peak at 0.77 ppm is assigned to the methyl (-CH_3) protons. Since each of the L, D, and T units contains a methyl group, the integrated intensity of this peak therefore represents the total number of all units. Based on the above analysis, the DB can be calculated using the following equation:

$$L = 0.47 \quad \text{(S2)}$$

$$D + L + T = 1.02 \quad \text{(S3)}$$

$$D + T = 1.02 - 0.47 = 0.55 \quad \text{(S4)}$$

$$DB = \frac{D + T}{D + L + T} = \frac{0.55}{1.02} = 0.54 \quad \text{(S5)}$$

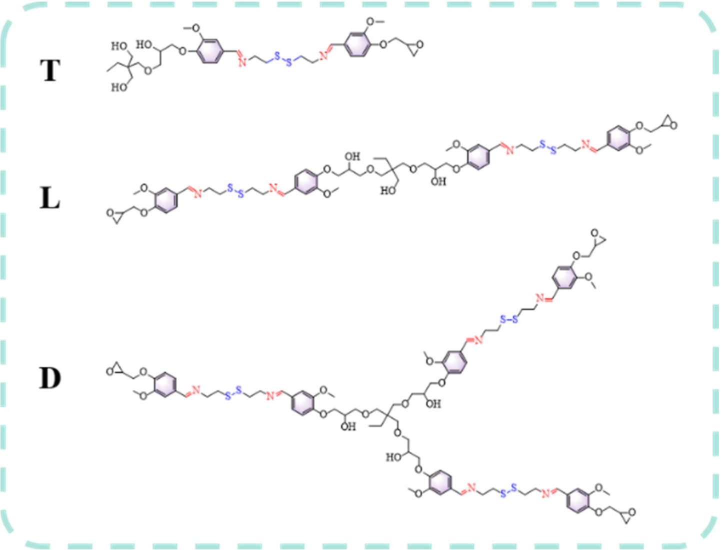

Figure S2. The dendritic, terminal, and linear units of HVT.

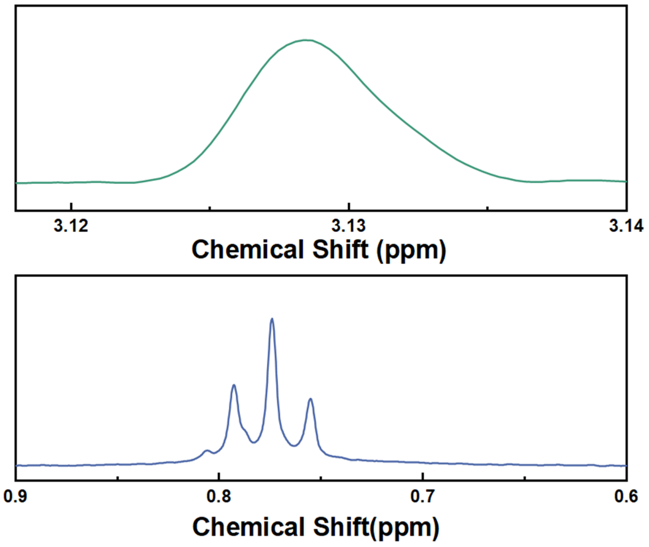

Figure S3. Partial ^{1}H NMR spectrum of HVT; (a) -CH_3, (b) -OH.

S3. Molecular weight of HVT

Table S1. Molecular weight and distribution of HVT.

Polymer	M_n (g·mol^{-1})	M_w (g·mol^{-1})	PDI
HVT	1500	2500	1.67

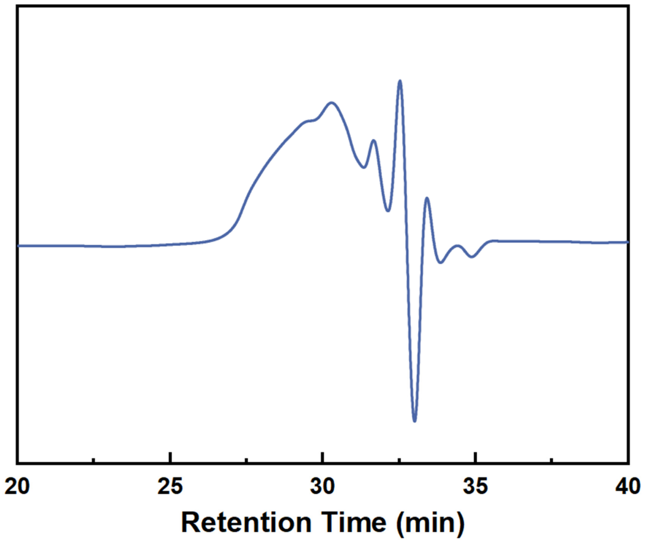

Figure S4. GPC curve of HVT.

S4. The curing dynamic of HVT/E51/VA

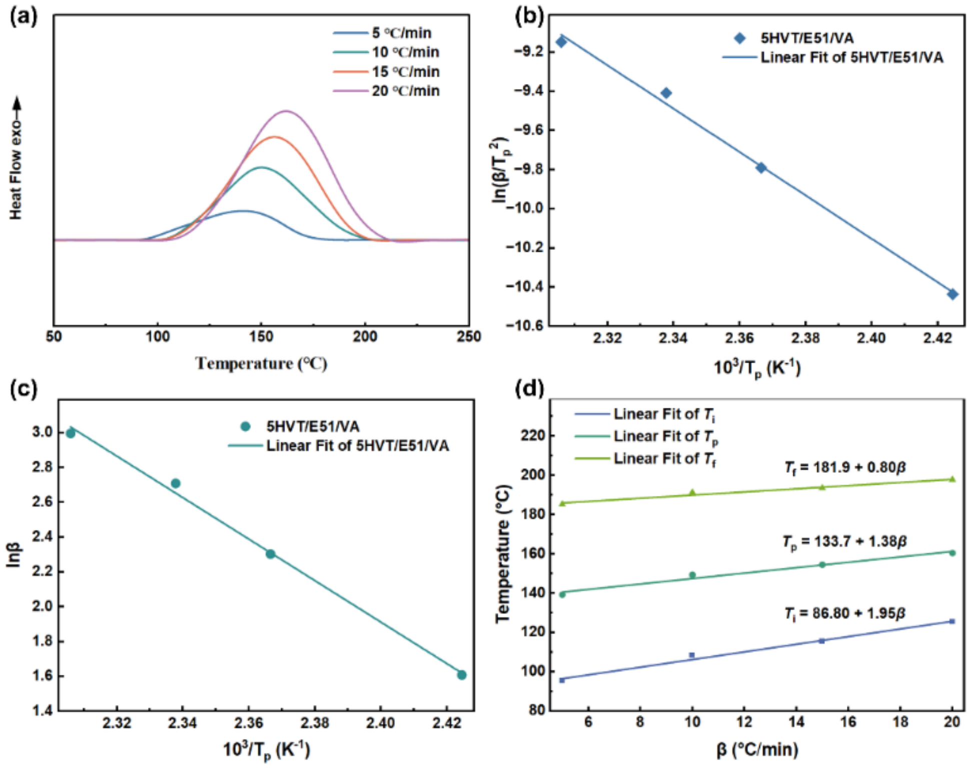

Figure S5. (a) DSC curves at different heating rates; the fitting curves for 5HVT/E51/VA based on (b) Kissinger and (c) Ozawa equations; (d) extrapolation curves of T-β.

The curing dynamic of 5HVT/E51/VA were investigated by DSC. The activation energy (E_a) is 91.53 kJ/mol and 94.12 kJ/mol, respectively, according to the Kissinger (S6) and Ozawa (S7) equations.

$$\ln\left(\frac{\beta}{T_p^2}\right) = \ln\left(\frac{AR}{E_a}\right) - \frac{E_a}{RT_p} \quad (S6)$$

$$\frac{d(\ln\beta)}{d\left(\frac{1}{T_p}\right)} = -\frac{1.052E_a}{R} \quad (S7)$$

where β represents heating rate (K/min), T_P denotes the curing exothermic peak temperature (K), A signifies the pre-exponential factor (min^{-1}), and R is the gas constant (8.314 J/mol·K).

The relationship between the heating rate β and the characteristic curing temperatures is displayed in Figure S5d. When β is zero, the theoretical gel temperature (T_i), theoretical curing temperature (T_P), and theoretical post-treatment temperature (T_f) of 5HVT/E51/VA is 86.8 °C, 133.7 °C, and 181.9 °C, respectively. Thus, the curing procedure is set as 90 °C × 3 h + 135 °C × 3 h + 185 °C × 3 h.

S5. Mass of HVT/E51/VA after immersion in different solvents.

Table S2. Mass of E51/VA and 5HVT/E51/VA before and after immersion in different solvents.

Solvents	E51/VA			5HVT/E51/VA		
	before immersion (g)	after immersion (g)	mass loss rates (%)	before immersion (g)	after immersion (g)	mass loss rates (%)
DMF	0.2233	0.2232	0.0448	0.3073	0.3071	0.0650
THF	0.2179	0.2178	0.0459	0.2716	0.2715	0.0368
DMSO	0.2083	0.2081	0.0960	0.2498	0.2496	0.0801
TCM	0.2300	0.2999	0.0435	0.2526	0.2525	0.0396
Act	0.2862	0.2860	0.0698	0.2374	0.2372	0.0842
EtOH	0.2577	0.2576	0.0399	0.2509	0.2508	0.0398
1M HCl	0.2396	0.2395	0.0417	0.2362	0.2360	0.0847
1M NaOH	0.2292	0.2291	0.0436	0.2515	0.2514	0.0397

S6. Paired t-test of E51/VA-R and 5HVT/E51/VA-R

This section may be divided by subheadings. It should provide a concise and precise description of the experimental results, their interpretation, as well as the experimental conclusions that can be drawn.

Table S3. Mechanical Properties and paired t-test of E51/VA-R and 5HVT/E51/VA-R.

Reprocessing cycles	Flexural strength (MPa)		Impact strength (kJ/m²)	
	E51/VA	5HVT/E51/VA	E51/VA	5HVT/E51/VA
0	74.3	124.4	12.98	20.16
1	69.2	116.8	14.07	21.04
2	68.3	116.1	11.91	18.92
3	59.9	103.8	10.60	17.68
Mean	67.9	115.3	12.39	19.45
Standard Deviation	6.1	8.5	1.47	1.51
95% Confidence Interval	[43.3, 51.4]		[6.09, 8.03]	
t(3)	36.9		153.48	
p-value	< 0.001		< 0.001	
